# A prospective, randomized, controlled trial of autologous haematopoietic stem cell transplantation for aggressive multiple sclerosis: a position paper

**DOI:** 10.1177/1352458512438454

**Published:** 2012-06

**Authors:** R Saccardi, MS Freedman, MP Sormani, H Atkins, D Farge, LM Griffith, G Kraft, GL Mancardi, R Nash, M Pasquini, R Martin, PA Muraro

**Affiliations:** 1 Hematology Department, Careggi University Hospital, Florence, Italy; 2 Department of Medicine (Neurology), University of Ottawa and the Ottawa Hospital Research Institute, Canada; 3 Department of Health Sciences, University of Genoa, Italy; 4 The Ottawa Hospital Blood and Marrow Transplant Programme, The Ottawa Hospital, Canada; 5 Internal Medicine Department, Saint Louis Hospital, Paris, France; 6 Division of Allergy, Immunology and Transplantation, National Institute of Allergy and Infectious Diseases, National Institutes of Health, Bethesda, USA; 7 Department of Rehabilitation Medicine, University of Washington, USA; 8 Department of Neuroscience, Ophthalmology and Genetics, University of Genoa, Italy; 9 Clinical Research Division, Fred Hutchinson Cancer Research Center, and University of Washington, USA; 10 Center for International Blood and Marrow Transplant Research, Medical College of Wisconsin, USA; 11 Department of Clinical Neuroimmunology and MS Research, Neurology Clinic, University Hospital Zürich, Switzerland; 12 Centre for Neuroscience, Imperial College London, UK

**Keywords:** autologous hematopoietic cell transplantation, prospective clinical trial, multiple sclerosis, consensus

## Abstract

**Background:**

Haematopoietic stem cell transplantation (HSCT) has been tried in the last 15 years as a therapeutic option in patients with poor-prognosis autoimmune disease who do not respond to conventional treatments. Worldwide, more than 600 patients with multiple sclerosis (MS) have been treated with HSCT, most of them having been recruited in small, single-centre, phase 1–2 uncontrolled trials. Clinical and magnetic resonance imaging outcomes from case series reports or Registry-based analyses suggest that a major response is achieved in most patients; quality and duration of response are better in patients transplanted during the relapsing–remitting phase than in those in the secondary progressive stage.

**Objectives:**

An interdisciplinary group of neurologists and haematologists has been formed, following two international meetings supported by the European and American Blood and Marrow Transplantation Societies, for the purpose of discussing a controlled clinical trial, to be designed within the new scenarios of evolving MS treatments.

**Conclusions:**

Objectives of the trial, patient selection, transplant technology and outcome assessment were extensively discussed. The outcome of this process is summarized in the present paper, with the goal of establishing the background and advancing the development of a prospective, randomized, controlled multicentre trial to assess the clinical efficacy of HSCT for the treatment of highly active MS.

## Introduction

**Figure 1.**
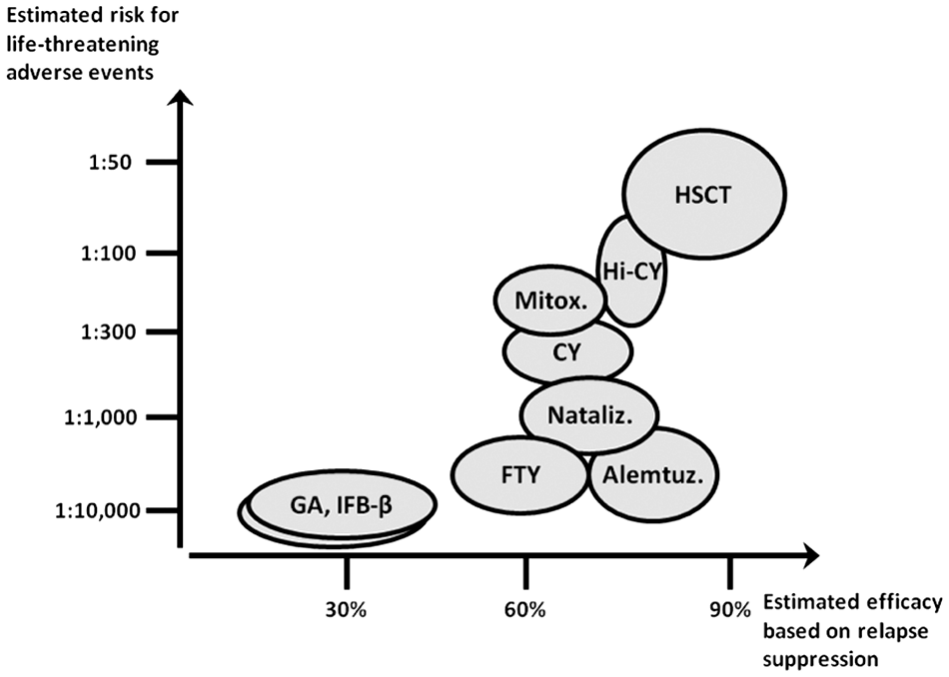
Estimated efficacy versus risks of approved and experimental therapies for MS. The plot indicates estimated, approximate risks vs. efficacy measures for some approved and experimental MS treatments. Estimated risks of life threatening unwanted effects are obtained from data available in the current literature.^39–50^ The percent relapse suppression observed in treated patients is presented as a surrogate of efficacy. The list of treatments is not meant to be exhaustive but to only include the agents that have immediate relevance for the HSCT trial design. Abbreviations: GA, glatiramer acetate; IFN-β, interferon beta; FTY, fingolimod; Mitox., mitoxantrone; CY, cyclophosphamide, Nataliz., natalizumab; Alemt., alemtuzumab, Hi-CY, high-dose cyclophosphamide, HSCT, autologous hematopoietic stem cell transplantation.

Current treatments of multiple sclerosis (MS) include high-dose corticosteroids for the treatment of exacerbations and long-term immuno-modulation/-suppression to reduce the number and severity of relapses, and slow down the progression of disability.^1^ The intensity of the immunosuppression is to some extent proportional to the risk of side effects and to the clinical response, expressed in terms of capacity to reduce the frequency of relapses (Figure 1).^2^ However, no disease-modifying agent (DMA) has been conclusively shown to significantly alter long-term outcomes; moreover, a subset of patients presents with aggressive disease which responds poorly to conventional treatments. Treatment alternatives are especially needed for this group of patients, i.e. those failing multiple treatments and rapidly accumulating clinical disability.

Haematopoietic stem cell transplantation (HSCT) is a well-established procedure for the treatment of poor-prognosis haematological malignancies.^3^ In the last 15 years HSCT has also been explored to treat patients with severe autoimmune diseases (AD) who were deteriorating despite receiving standard treatments.^4^ The rationale is derived both from experimental models^5^ and from the observations of positive effects on a coincidental AD of transplants given for a conventional haematological indication.^6^

Autologous HSCT for MS has been repeatedly and recently reviewed.^7^ Most patients were treated in small phase 1–2 trials, either single-^8–15^ or multi-centric;^16,17^ currently no data are available from prospective comparative trials. Two single-centre, retrospective analyses of long-term outcome were recently published and both showed a sustained progression-free survival (PFS) beyond 5 years after HSCT.^15,18^ The neurological outcome was considerably more favourable in patients transplanted in the relapsing–remitting (RR) phase^18^ and/or showing an inflammatory pattern at magnetic resonance imaging (MRI) during pre-transplant screening.^15^ Indeed, case series and reports of excellent outcome in particularly aggressive forms of MS^19,20^ support the notion of efficacy of HSCT in MS patients with prominent inflammatory activity. The risk of treatment-related mortality (TRM) in HSCT, conventionally perceived to be unacceptably high, has decreased since 2001 to 1.3%, according to an analysis of the European Blood and Marrow Transplantation Group (EBMT, www.ebmt.org) Registry,^7^ likely due the avoidance of aggressive regimens which resulted in frequent toxicity. In particular, the use of busulfan was found to be significantly associated with higher TRM in multivariate analysis,^21^ and high doses of rabbit anti-T-lymphocyte globulin (ATG), associated with ex-vivo T-cell depletion and an intense conditioning regimen (CR), resulted in unexpected occurrence of EBV-associated lymphomas.^22^ In 2004, the EBMT launched a prospective, randomized phase 2 trial (ASTIMS, www.astims.org) comparing autologous HSCT with mitoxantrone, with new MRI lesions as the primary endpoint (number of new T2 lesions at 1 and 2 years after the randomization). In total, 21 patients were randomized to the two trial arms and the follow-up analysis is currently ongoing. Two phase 2, single-arm trials were carried out in the USA (www.halt-ms.org; or www.clinicaltrials.gov, id. NCT00288626) and Canada (www.clinicaltrials.gov, id. NCT01099930), respectively; both trials closed accrual in 2010. The Canadian trial has reported results in preliminary form showing complete suppression of relapses and of new MRI lesions in 23 evaluable patients over a median follow-up of 5 years.^12^ The US HALT-MS trial preliminary results in 25 patients with highly active, treatment-refractory RRMS have reported a 77% event-free survival at 2 years post-HSCT utilizing a stringent composite endpoint that included relapses, new MRI lesions and progression of neurological disability.^23^

The cumulative experience described above is highly suggestive of a strong treatment effect of HSCT on inflammatory manifestations of MS, but the efficacy and safety of HSCT compared with currently available therapies has not yet been established. In November 2008 a meeting of experts in MS and bone marrow transplantation was held in Minneapolis under the auspices of Center for International Blood & Marrow Transplant Research (CIBMTR), the National Institute of Allergy and Infectious Diseases (NIAID), and the EBMT in order to establish shared criteria for the assessment of efficacy of HSCT in MS and evaluate the feasibility of a new prospective, multicentre trial.^24^ One year later EBMT and NIAID supported an international meeting in Florence, aimed to update knowledge and strategies of HSCT for AD. Panels of specialists were appointed for each major AD (www.adflorence.org) and the MS panel included neurologists, haematologists and statisticians from both Europe and North America.^25^ There was uniform agreement about the need for a prospective, controlled trial aimed at investigating the role of HSCT in the treatment of aggressive MS. This report describes the progress to date of this interdisciplinary group of specialists in the design of such a trial.

## Choice of trial

**Figure 2.**
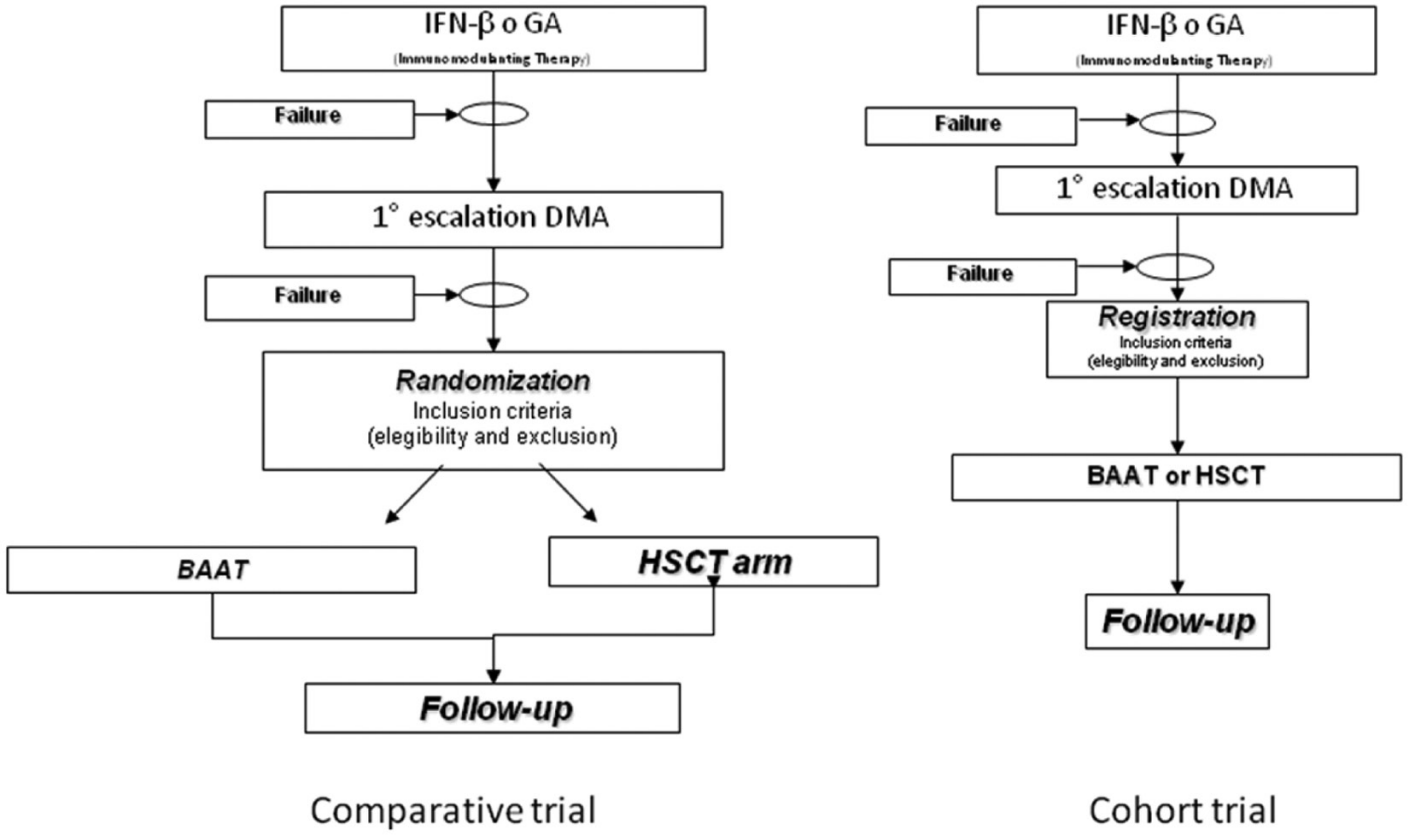
Prospective clinical trials for HSCT in MS. Choice of trial design. BAAT, best available and approved treatment; DMA, disease-modifying agent; HSCT, haematopoietic stem cell transplantation.

Two approaches to a prospective trial were considered (Figure 2): a cohort trial, where patients fulfilling homogeneous criteria of disability and unsatisfactory response to approved treatments are registered, treated according to the policy of each participating centre (including the transplantation option) and prospectively followed-up. This approach would likely find higher acceptance and easier implementation; however, it would be expected to add little knowledge to the existing data. Therefore, all panellists agreed that a large, international, phase 3, randomized trial with a clinical endpoint is currently needed. This should be a phase 3 study where patients are randomized between autologous HSCT and the appropriate non-HSCT comparator.

## Non-HSCT comparator

There was considerable discussion regarding the most appropriate comparator. Although some discussants favoured a single agent (e.g. natalizumab), there were prevailing concerns that some agent/s might not be available in all participating countries. Approval of an additional 3–4 new therapies for RRMS is foreseen in the next 2 years,^1^ with differences in their introduction expected in different countries due to regulatory issues. Furthermore, there is no standard of therapy for patients failing approved treatments for MS who are eligible for HSCT, therefore they are treated differently according to centre policy and local regulations on drugs and reimbursement policies. As a result of these considerations, a consensus view emerged that the trial should be designed with randomization between autologous HSCT and Best Available and Approved Treatment (BAAT). The BAAT is defined as, and will consist of, the treatment available as licensed therapy to the individual patient in his/her country of residence and approved under the applicable healthcare scheme.

## Trial goals

- To establish the safety and efficacy of autologous HSCT in comparison with approved therapies in a specific subset of highly active RRMS;
- To address questions related to the immunopathogenesis of MS by accompanying mechanistic studies;
- To create a comprehensive repository of biosamples for planned and future mechanistic studies.

## Inclusion criteria

The definition of inclusion criteria was felt to be a key point. The aim of the panellists was to capture a subset of patients with relatively early MS who are at high risk of suffering early disease progression based on their high inflammatory activity. Prognostic predictions are difficult in MS, and three domains were considered important to formulate criteria that select subjects at greater risk of poor prognosis and potential eligibility for the trial: (1) clinically significant relapses; (2) MRI activity; and (3) treatment failure. The following specific criteria were selected:

- Patients aged 18–45 years with Expanded Disability Status Scale (EDSS) score between 2.5 and 5.5, who are within 5 years from initiation of the first therapy with DMAs and who fulfil the criteria for highly active RRMS within the past 2 years.
  ○ The definition of ‘highly active’ RRMS includes:
    ■ ≥1 severe relapses (ΔEDSS ≥1 and Fatigue Severity Scale (FSS) of ≥2 in motor, cerebellar or brain stem deficit (or documented changes in neurological examination consistent with these magnitudes) and/or incomplete recovery from clinically significant relapses;
      **and**
    ■ ≥1 gadolinium-positive (Gd+) lesion of diameter ≥ 3 mm **or** accumulation of ≥ 0.3 T2 lesions/month in two consecutive MRI 6–12 months apart.
  ○ Patients are eligible after failure of conventional treatment.

The upper limit of 45 years was chosen for the following reasons: (1) the low probability of older patients to fulfil the inclusion criteria; (2) their faster shift to the secondary progressive phase of disease;^26^ and (3) one report of a worse outcome after HSCT in patients aged over 40 years.^21^

○ Patients must have failed at least one and up to three subsequent lines of BAAT. Treatment failure is defined as follows:
  ■ Evidence of relapses, increased EDSS (by 0.5 for any EDSS ≥5.5 or 1.0 for any EDSS <5.5, confirmed after 3 months,^27^ continuing Gd+ or new T2 lesions ≥6 months from starting therapy (same as ‘highly active’).
○ Patients will be given the option to be randomized after failing only first-line therapy, which may have been either an immunomodulating or immunosuppressive therapy, (e.g. natalizumab, fingolimod, IFN-β, glatimer-acetate).

**Figure 3.**
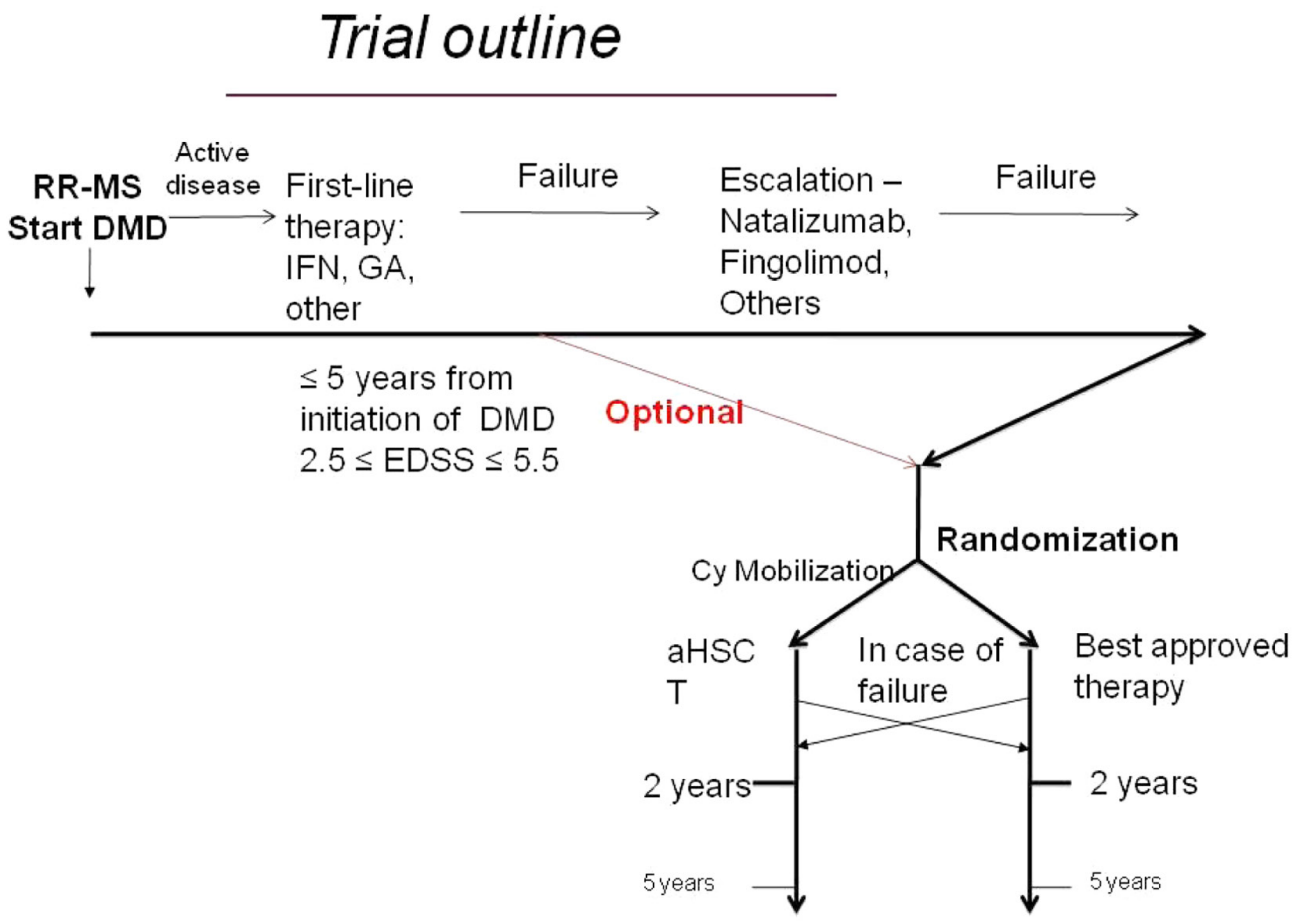
Prospective, controlled trial for HSCT in a multiple sclerosis. Trial design.

This last item was extensively discussed since some neurologists consider failure of only first-line therapy as ‘too early’ to randomize patients to HSCT. Others consider the above criteria for ‘highly active’ MS as an indicator of poor prognosis, therefore the randomization at this stage being appropriate. Taking all these considerations into account, it was decided to allow, but not to require, the inclusion of patients after a first-line treatment failure only. Patients will be stratified according to the treatments administered before the randomization, should numbers permit (see Figure 3).

The informed consent process should include a detailed discussion of the treatment risks and non-trial treatment alternatives. Patients must be adequately screened to exclude coincidental organ failure, to minimize the transplant-associated toxicity.

Most of the MS panellists estimated that 8–10 patients fulfilling the inclusion criteria present each year at their respective institutions (~5% of patients in dedicated MS centres with a large base of annual referrals).

## Outcomes of the study

A major response should be expected by an aggressive treatment involving significant risk such as HSCT. Such response could be defined as PFS lasting 5 years free from further immunosuppressive treatment. A long-term follow-up, such as the proportion of patients still progression-free at a given interval (e.g. 3 or 5 years), would greatly increase the discrimination power between the two treatment arms; however, there was consensus that a follow-up duration longer than 2 years before assessing the primary outcome would not be feasible for the core trial. In the ASTIMS trial, randomization prior to HSCT hampered recruitment, due to the rapidly evolving clinical course of patients; this concern should be carefully considered in the trial design. For this reason, we favoured a ‘time to event’ endpoint, allowing patients initially randomized to BAAT who continue to deteriorate to cross-over to HSCT once they have reached the ‘time to’ endpoint.

- The primary outcome is the time to treatment failure, compared between treatment arms.
  ○ Definition of treatment failure is the occurrence of a severe relapse (see above) or sustained EDSS worsening (i.e. increase by 0.5 for any EDSS ≥5.5 or 1.0 for any EDSS <5.5, confirmed after 3–6 months), or a new (Gd+) lesion ≥3 mm or accumulation of >0.3 mm T2 lesions/month in two consecutive MRI 6–12 months apart.

Patients reaching the primary endpoint will be given the chance to receive the treatment of the other arm (i.e. HSCT in case of failure of the control treatment; and vice versa).

This primary endpoint optimizes the trial design, in that it requires a shorter follow-up as compared with a 2-year trial based on a relapse rate endpoint, and preserves a similar statistical power, especially in case of early post-treatment treatment failure events. The option to cross-over for patients reaching the endpoint will facilitate the availability of patients for a randomized trial.

Secondary objectives will include:

○ Comparison of overall survival;
○ Treatment-related mortality;
○ MRI outcomes including lesion and atrophy metrics, to be defined;
○ Rates of disease progression;
○ Rate of adverse events, infectious complication;
○ Quality of life;
○ Improvement of disability;
○ Response to post-failure treatment between treatment arms; and
○ Need for any MS-related treatment.

## Sample size

We calculated that 114 patients (57 in each group) are required to provide a power of 90% (at a confidence level of 5%) to detect a clinically meaningful relative reduction in the actuarial cumulative probability to have a failure over 1 year of 50% (corresponding to a hazard ratio (HR)=0.44) in the HSCT group as compared with the BAAT group.

Sample size was calculated by using a log-rank test on the assumption that 40% of failures would occur in the BAAT group at 1 year, with an exponential distribution for time to failure, with an accrual time of 1 year and a follow-up of 2 years.

## Transplant methodology

**Figure 4.**
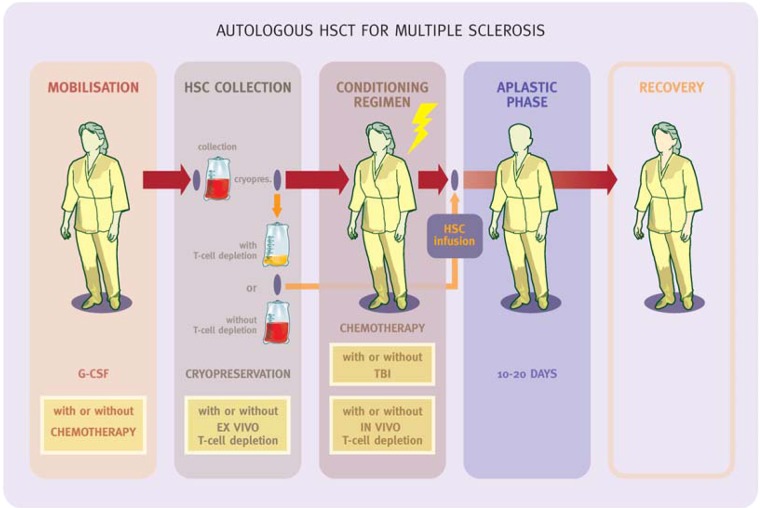
Transplant methodology. The boxes represent variables related to the intensity of the treatment. (from Saccardi R and Gualandi F. *Autoimmunity*, 2008; 41(8): 570-576.)

The intensity of the immunosuppression delivered by the transplant treatment is dependent upon several factors:

1. The use of chemotherapy, such as cyclophosphamide (Cy) in the stem cells mobilization regimen;
2. *Ex-vivo* T-cells depletion (purging);
3. An intense CR;
4. The inclusion of ATG or monoclonal antibodies (i.e. alemtuzumab) in the CR to maximize the immunosuppression (Figure 4).

**Figure 5.**
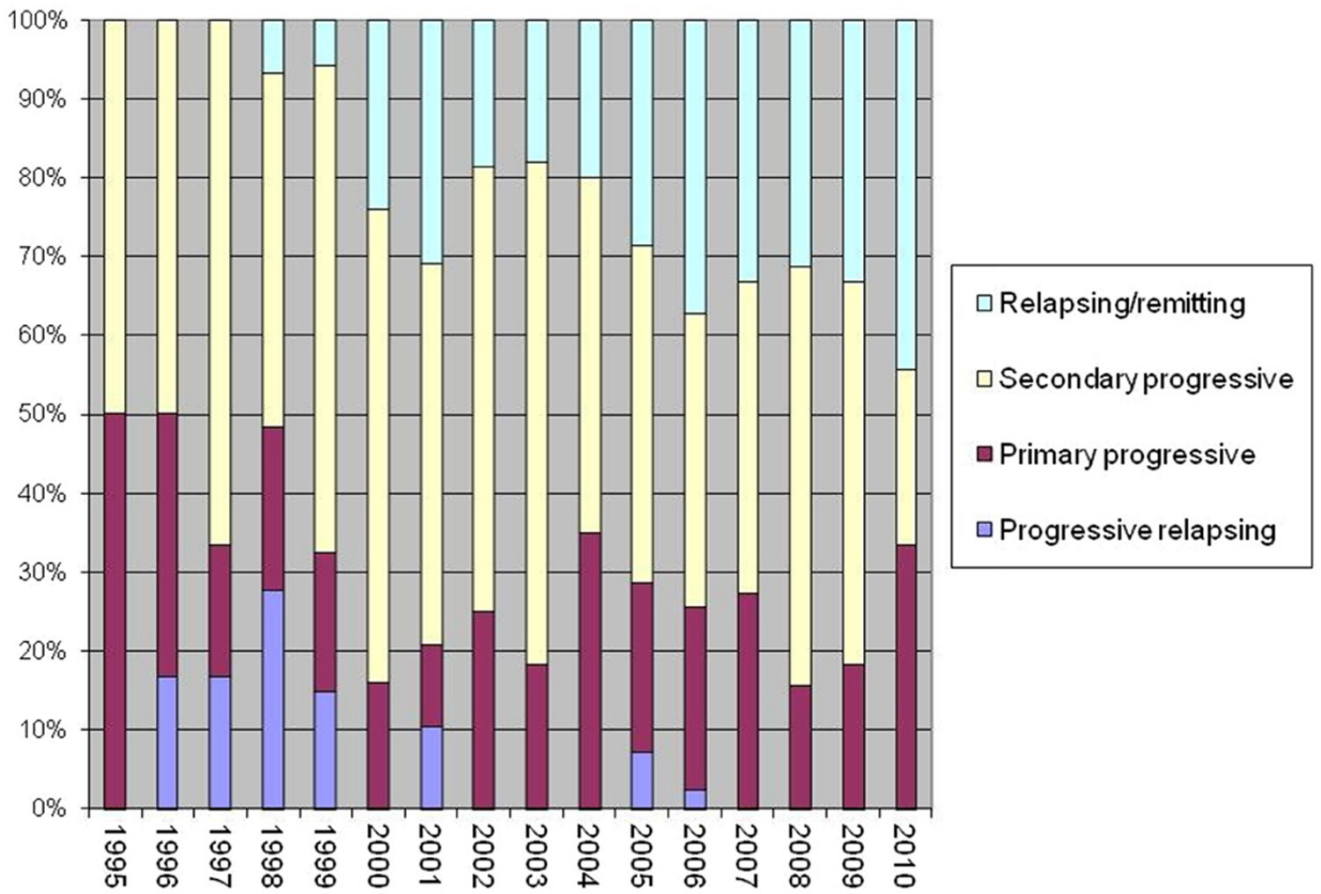
Proportion of multiple sclerosis forms at transplant baseline across the years. EBMT Registry data.

The overall intensity of the treatment can be modulated by changing the combination of such variables used (from none to all of them). The initial experiences of autologous HSCT for MS were dominated by high-intensity regimens as a high rate of relapse was expected, following some initial reports in ADs.^28^ With time, toxicity of such regimens became a clinical issue,^21,22,29^ and at the same time evidence emerged that less aggressive treatments have a favourable risk/benefit profile, as recently reviewed.^30^ Indeed, the importance of the disease phase (RRMS vs. secondary progressive MS) over the intensity of CR in determining the outcome was increasingly perceived by the teams involved in transplant programmes,^15,18^ and this is reflected by the gradual increase in RRMS cases treated being reported to the EBMT Registry over the years (Figure 5).

The most widely used CR in Europe for MS is the intermediate-intensity BEAM/ATG regimen (Carmustine, Etoposide, ARA-C and Melphalan in combination with polyclonal rabbit ATG).^21^ This regimen is still widely used due to the good safety/efficacy profile in lymphoproliferative diseases. BEAM has more recently been included in MS trials in Asia^31^ and in the USA (www.halt-ms.org). A lower-intensity (non-myeloablative) regimen, based on Cy and ATG or alemtuzumab, was also reported from Burt and colleagues at Northwestern University^32^ in a group of patients with early RRMS. The analysis of outcomes showed a low toxicity, but a trend to a higher frequency of relapses or recurrence of MRI activity (23%) than in higher-intensity CRs, although patients were reported to respond well to further immunotherapies. A prospective comparative trial would be needed to draw conclusions about its safety and efficacy profile.

○ Mobilization of stem cells
  • Patients will be mobilized by the administration of cyclophosphamide, 4 g/m^2^ followed by G-CSF, 10 mcg/kg. Peripheral blood stem cells (PBSC) will be collected by continuous flow leukapheresis, targeted to cryopreserve ≥3×10^6^ CD34^+^ cells/kg.

The combination of Cy and G-CSF is preferred because

1. Most patients improve after mobilization that includes a standard (2–4 g/m^2^) Cy regimen;^17^
2. Cy reduces the potential risk of MS exacerbation in response to G-CSF;^33^
3. Inclusion of Cy in the mobilization regimen decreases the number of T cells in the apheresis collection, possibly obviating the need for ex-vivo T-cell depletion;
4. Although comparative data are not available, no post-transplant carryover of T-cell clones was shown to have originated from a Cy-mobilized CD34-selected hematopoietic graft.^34^
  ○ Graft manipulation
    ■ PBSC will be cryopreserved unmanipulated.

Ex-vivo T-cell depletion techniques such as CD34^+^ selection were proven to be ineffective in a prospective comparative trial for rheumatoid arthritis^35^ and in a retrospective analysis of the MS EBMT database.^21^ Moreover, the feasibility of the study would be improved and transplant costs would be reduced by using unmanipulated grafts.

○ CR
  ■ ‘BEAM’ regimen (BCNU 300 mg/m^2^ on day -6, cytosine arabinoside, 200 mg/m^2^ and etoposide 200 mg/m^2^ day -5 to day -2, melphalan 140 mg/m^2^ day -1) + peri-transplant ATG is preferred by most of the transplanters as an intermediate-intensity regimen.

BEAM+ATG is the most commonly used regimen in Europe, and it showed a satisfactory toxicity profile both in the EBMT Registry analysis^21^ and in the North American Halt-MS trial.^36^ Low-intensity regimens (i.e. Cy+alemtuzumab or ATG) do not seem likely to achieve the goal of 5 years of disease and treatment-free survival, when considering the only prospective trial reported so far.^32^ A Brazilian prospective study compared BEAM/ATG vs. Cy/ATG, reporting a similar outcome but a lower toxicity in the latter. However, the patients included in both groups all had advanced disease, which limited the usefulness of the comparison; also, a high dose of horse ATG was administered in the BEAM group, and may have caused the high observed toxicity (TRM 7.5%) which exceeds any other report.^37^ Finally, high-intensity regimens (i.e. busulphan + Cy) can also be considered; it is thought that toxicity might be higher, but with the advent of intravenous formulations of busulphan and monitoring of blood levels, this might be minimized, as reported in the Canadian trial.^12^ All the participants agree that data from the Canadian trial are highly promising. However, the choice of the CR for a large, prospective, multicentre trial needs to be based on published data either from prospective studies and/or from Registry analysis, and therefore BEAM was considered the best choice at the current time. New published data on the CR might change the current preference.

## Immunological studies and repository of biosamples

The demonstration by Muraro et al.^38^ that autologous HSCT indeed leads to extensive renewal of the T-cell repertoire provided crucial evidence to document that autologous HSCT goes beyond a profound and long-lasting immunosuppression, which can be achieved by conventional treatments. However, many questions remain regarding the therapeutic mechanisms of autologous HSCT, and these should be examined during the prospective phase 3 trial. Therefore, a comprehensive repository of biomaterials should be created in order to allow interested investigators to address important questions in parallel with the trial and later. An assessment of immune reconstitution will be integrated in the trial and will be carried out on freshly collected blood specimens. A policy will regulate the utilization of the stored biological samples for qualified research projects, and its implementation will be overseen by a specific study committee.

## Logistics and funding

The target population of the trial constitutes a relatively small subset of all patients with RRMS, and in order to meet patient accrual within a period of 2 years a large number of countries and participating centres need to be involved. Currently a clear sign of interest has been received from groups in Canada, France, Germany, Italy, Switzerland, the USA, and the UK. Other centres in Brazil, Spain, and the Czech Republic might follow. Each centre will need an established team of MS neurologists and bone marrow transplanters, as well as access to facilities including MRI and a laboratory infrastructure for the collection, characterization and storage of the biological samples for research. Criteria will be established to select centres with the required capacity and experience. Nursing care will require interaction between neurological and haematological nursing staff. A comprehensive plan for data collection, management, quality control and analysis for this complex international study will need to be developed.

Conducting a phase 3 controlled multi-centre trial comparing a treatment such as autologous HSCT with other treatments will not only be a major organizational effort, but will also be expensive. We do not expect a high level of interest in autologous HSCT for treatment of MS from pharmaceutical companies, which may even consider the treatment as market competition. Therefore, funding will have to be sought from public funding bodies and charitable foundations. A strategy for funding should consider and coordinate public resources at the international (NIH, EU FP-7 or later framework programmes), national (MS societies, national research foundations, governmental funding calls, others), and local (at the respective centres, their universities, cities or states) level.

## Conclusions

We believe that the proposed outline protocol represents a solid foundation for the development of a randomized controlled phase 3 trial of HSCT for MS. Interested neurologists, bone marrow transplanters and MS patient associations are invited to engage in the final stages of protocol development and in the trial planning.
